# Caso de hernia interna abdominal a través del hiato de Winslow en urgencias de pediatría

**DOI:** 10.23938/ASSN.1068

**Published:** 2024-03-07

**Authors:** David Del Cerro Rodríguez, Samuel González-Pola Yuncal, Santiago Altamirano, Silvia García Saavedra, Manuel Gómez Tellado

**Affiliations:** Servicio Gallego de Salud Complejo Hospitalario Universitario de A Coruña Servicio de Cirugía Pediátrica A Coruña España

**Keywords:** Hernia del Foramen de Winslow, Obstrucción Intestinal, Cirugía, Pediatría, Hernia Foramen, Winslow, Intestinal Obstruction, Surgery, Pediatrics

## Abstract

Las hernias internas abdominales son una causa poco frecuente de obstrucción intestinal en las urgencias de pediatría, siendo excepcional la herniación a través del hiato de Winslow (menos del 0,5% de las hernias).

Presentamos el caso clínico de un varón adolescente de 15 años sin antecedentes quirúrgicos previos, con clínica de dolor abdominal y vómitos, en el que la tomografía computarizada sugería un cuadro de obstrucción intestinal a causa de hernia interna a nivel del hiato de Winslow. Precisó de intervención quirúrgica mediante laparoscopia exploradora, reconvertida por mala visualización a laparotomía media supraumbilical, para reducción del asa de íleon herniada. Esta presentaba buen aspecto y no fue necesaria la resección intestinal. No se realizó ninguna técnica preventiva para disminuir el riesgo de recidiva. Postoperatoriamente, el paciente presentó una colección pélvica manejada de manera conservadora con antibióticos. Actualmente se encuentra en seguimiento en consultas externas de cirugía pediátrica.

## Abstract (en)

Abdominal internal hernia is a rare cause of intestinal obstruction in pediatric emergency departments, being the herniation through the foramen of Winslow an exceptional entity (less than 0.5% of the herniae).

We report the case of a 15-year-old adolescent male without previous surgical interventions who presented with abdominal pain and vomiting; computed tomography scans showed intestinal obstruction due to an internal hernia through the foramen of Winslow. To reduce the herniated ileum, the patient required surgical intervention with diagnostic laparoscopy, which, due to bad visualization, was changed to supraumbilical midline laparotomy. There was no need to resect the affected ileum as it appeared healthy. We did not perform a preventive technique to reduce the risk of recurrence. Postoperative pelvic collection was conservatively managed with antibiotics. The patient undergoes regular follow-up in the pediatric surgery department.

## INTRODUCCIÓN

Las hernias internas abdominales son un una patología muy poco frecuente, especialmente durante la edad pediátrica, causando el 0,5-6% de los casos^1^. Solo el 8% corresponden a hernias por el hiato de Winslow^2^, con menos de 200 casos publicados. El hiato de Winslow, o foramen epiploico, representa la única comunicación entre la cavidad peritoneal mayor y la transcavidad de los epiplones. Tiene unos tres centímetros de diámetro y está limitado anteriormente por el ligamento hepatoduodenal y vena porta, posteriormente por la vena cava inferior, teniendo como borde superior el lóbulo caudado del hígado y la primera porción del duodeno como borde inferior^1^. En condiciones normales, este hiato permanece colapsado debido a la presión intra-abdominal.

La víscera más frecuentemente implicada es un asa de intestino delgado o ciego, siendo excepcional la implicación de otros tramos de intestino u otras vísceras abdominales^1^. La forma de presentación suele ser inespecífica, con un cuadro compatible con obstrucción intestinal, con dolor abdominal epigástrico, distensión y vómitos^3^. Aunque la radiografía simple de abdomen puede arrojar alguna pista acerca del diagnóstico, resulta fundamental la realización de una tomografía computarizada para tratar de filiar la causa. No obstante, en la mayoría de los casos no se obtiene el diagnóstico definitivo hasta evidenciar los hallazgos intraoperatorios, siendo la cirugía el estándar oro del tratamiento.

El objetivo del presente artículo es describir esta entidad infrecuente, especialmente en urgencias de pediatría, para que el profesional encargado de su manejo la incluya en su diagnóstico diferencial ante hallazgos clínicos y radiológicos similares, disminuyendo así su comorbilidad al no retrasar el diagnóstico.

## CASO CLÍNICO

Presentamos el caso clínico de un varón de quince años, sin antecedentes médicos ni intervenciones previas, que acude al servicio de urgencias de pediatría con clínica de vómitos y dolor abdominal epigástrico de cuatro días de evolución, manteniéndose afebril durante la evolución del cuadro.

Inicialmente fue manejado como gastroenteritis, pero ante la ausencia de mejoría, con persistencia del dolor abdominal de tipo cólico a nivel epigástrico y de los vómitos de aspecto bilioso, acude a urgencias para nueva valoración.

**Figura 1:**
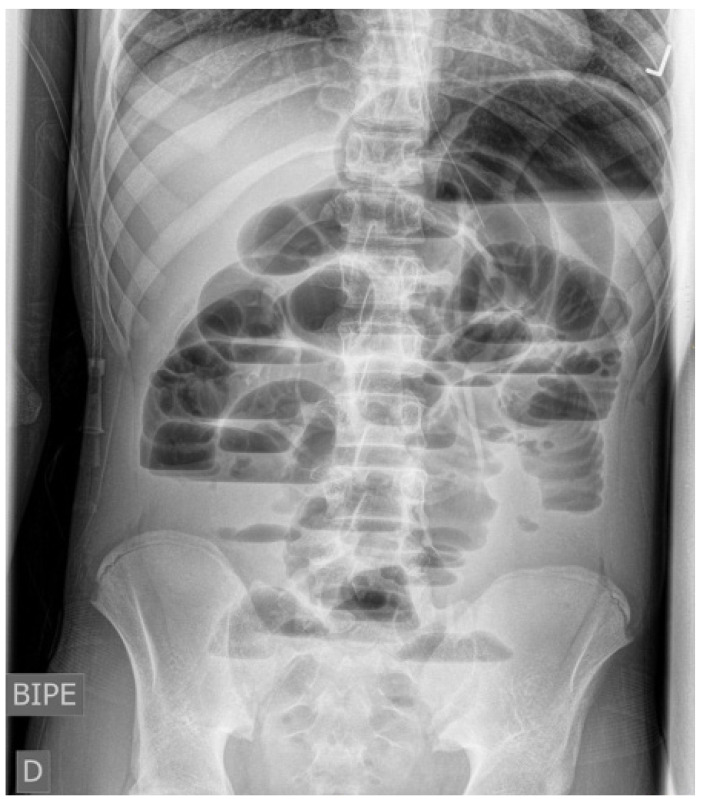
Radiografía simple de abdomen. Distensión de múltiples asas de intestino delgado con niveles hidroaéreos compatible con obstrucción de intestino delgado. No se visualiza ciego en fosa

A la exploración física el paciente presentaba aceptable estado general, se encontraba afebril, con signos leves de deshidratación. El abdomen se encontraba distendido, sin datos de peritonismo y con ruidos hidroaéreos disminuidos. La analítica no presentaba hallazgos significativos, realizándose una radiografía de abdomen con hallazgos sugestivos de obstrucción intestinal (Fig. 1).

**Figura 2:**
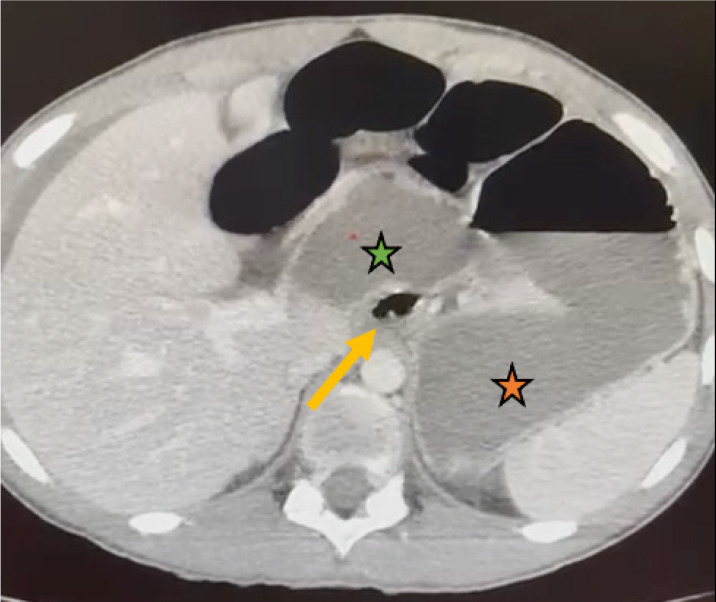
Tomografía computarizada. Presencia de asa intestinal en el inicio de la transcavidad de los epiplones (flecha amarilla) que parece continuarse a través del hiato de Winslow. Estómago (estrella naranja) y duodeno (estrella verde).

Dada la evolución se decide realizar una tomografía computarizada urgente, en la que se observó presencia de ascitis e importante dilatación de asas de intestino delgado, sugiriendo la interposición de un asa de intestino delgado en el inicio de la transcavidad de los epiplones, con cambio de calibre a nivel del hiato de Winslow (Fig. 2).

**Figura 3:**
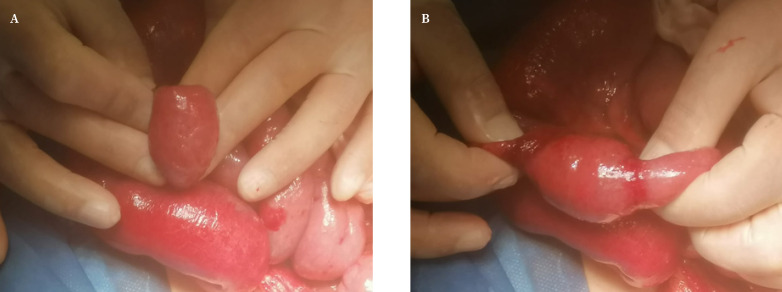
Vista intraoperatoria del tramo del asa de íleon herniada a través de hiato de Winslow. **A**. Cambio de calibre del asa a nivel del hiato. **B.** Impronta congestiva a nivel distal y proximal.

Se realizó intervención quirúrgica urgente, realizando inicialmente una laparoscopia exploradora. Se observaban asas de intestino delgado dilatadas, e íleon terminal, ciego y colon ascendente de calibre normal pero localizados en hipocondrio derecho, con el ciego muy móvil y sin presentar adherencias al espacio parietocólico derecho. Siguiendo proximalmente el íleon terminal, se observaron asas de intestino delgado de distinto calibre desde la profundidad de la teórica localización del hiato de Winslow. Se consiguió traccionar de ciego e íleon terminal hasta desplazarlos a fosa iliaca derecha, pero sin llegar a identificar correctamente el punto de cambio de calibre, ya que la interposición del borde inferior del hígado y la distensión de asas de intestino delgado dificultaban la técnica. Se intentó mejorar la visualización mediante punción percutánea de un asa dilatada para vaciar el gas, sin mejoría. Para asegurar la resolución del cuadro obstructivo se decidió realizar una laparotomía media supraumbilical. Al acceder a cavidad se evidenciaba el cambio de calibre en íleon, a unos 40 centímetros de la válvula ileocecal, con signos compatibles con herniación de un tramo de unos cinco centímetros de íleon a través de hiato de Winslow. En ambos extremos del asa herniada observamos la impronta congestiva del hiato sobre el asa (Fig. 3). Se identificó el hiato de Winslow, de calibre normal, por lo que no se realizó ninguna técnica preventiva para disminuir el riesgo de recidiva.

Durante los primeros días del postoperatorio el paciente presentó un íleo paralítico, pudiendo retirar la sonda nasogástrica a los cinco días de la cirugía e iniciando tolerancia oral con buena respuesta. Simultáneamente desarrolló una colección pélvica que se manejó de forma conservadora con antibióticos, con buena evolución. Finalmente, el paciente fue dado de alta a los diez días de la intervención, con tránsito conservado, manteniéndose afebril y con buen estado general.

Actualmente el paciente está en seguimiento en consultas externas de cirugía pediátrica. Tanto el paciente como su familia firmaron la autorización para la publicación de este caso clínico.

## DISCUSIÓN

Las hernias internas abdominales son una patología muy poco frecuente, representando entre el 0,2 y el 0,9% de todas las hernias^4^, y entre el 0,5 y el 6% de todas las causas de obstrucción intestinal^1^. Dentro de este grupo de patologías, encontramos hernias paraduodenales (50-55%), pericecales (10-15%), transmesentéricas o transmesocólicas (8%), pélvicas o perivesicales (6%), sigmoides (6%) o transomentales (1-4%)^4^. De todas ellas, la hernia por el hiato de Winslow representa el 8%^1^^,^^5^.

En el 60-70% de los casos de hernia por el hiato de Winslow la víscera herniada es un asa de intestino delgado, al igual que en el caso que presentamos. Encontramos englobado el ciego y el colon ascendente en el 25-30% de los casos, siendo excepcional la implicación del colon transverso o de la vesícula biliar^6^ o, como en el caso publicado por Welaratne y Nasoodi, que la hernia comprima la vía biliar extrahepática^7^, causando síntomas de ictericia obstructiva al paciente.

Los hallazgos clínicos suelen ser inespecíficos, presentándose en la mayoría de los casos como un dolor cólico epigástrico, con signos sugestivos de obstrucción intestinal^8^, como en el caso de nuestro paciente.

La radiografía simple de abdomen no suele aportar información relevante más allá de la posible existencia de una obstrucción intestinal. No obstante, en algunos casos pueden identificarse algunos hallazgos sugestivos esta patología, como presencia de asas intestinales en el cuadrante superior izquierdo del abdomen y mediales a la curvatura menor del estómago, desplazamiento inferior del colon transverso y ausencia del ciego en la fosa iliaca derecha. De todas ellas, en la radiografía de nuestro paciente se apreció la ausencia de ciego en cuadrante inferior derecho, hecho que se comprobó posteriormente en la intervención quirúrgica al encontrar un ciego muy móvil, sin adherencias a la pared, en el hipocondrio derecho.

La tomografía computarizada puede ser de gran ayuda para caracterizar la entidad previamente a la generalmente indispensable intervención quirúrgica. Los hallazgos típicos en la tomografía computarizada como dato sugestivo de herniación de íleon terminal, ciego o colon ascendente son la presencia de un asa intestinal por detrás del pedículo hepático, la compresión de la vena porta, la presencia de múltiples asas de intestino en la región subhepática desplazando el estómago anterolateralmente, y la ausencia de ciego en la fosa ilíaca derecha^8^. No obstante, dada la rareza y los hallazgos inespecíficos del cuadro, no es infrecuente que el diagnóstico definitivo se alcance intraoperatoriamente, lográndose el diagnóstico preoperatorio en tan solo el 10% de los casos^9^. En el caso que presentamos, el diagnóstico sí fue preoperatorio, gracias al asa visualizada en la entrada de la transcavidad de los epiplones.

Los factores predisponentes pueden dividirse en congénitos y adquiridos. Los congénitos están relacionados con un hiato más laxo y de mayor diámetro, mesenterio redundante, o mayor movilidad del intestino delgado por falta de adherencias a la pared abdominal posterior. Los adquiridos tienen relación con un aumento de presión intraabdominal, acompañada del desplazamiento craneal de las asas intestinales, como ocurre en el embarazo^10^. Los pacientes a los que se ha realizado una colectomía subtotal también tienen más riesgo por la tendencia del intestino delgado a localizarse en el epigastrio^11^; también se han asociado casos a la funduplicatura de Nissen o la colecistectomía laparoscópica^12^. Nuestro paciente no presentaba ningún antecedente quirúrgico de interés.

La intervención quirúrgica es el tratamiento de elección, sirviendo al mismo tiempo de método diagnóstico en la mayoría de los casos. La reducción de la hernia puede llevarse a cabo de manera laparoscópica o mediante laparotomía. El 30% de los casos descritos entre los años 2017 y 2021 se solucionaron mediante laparoscopia, sin necesidad de reconversión, según la revisión realizada por Huang y col^13^.

En función de la afectación del asa herniada puede ser necesaria la resección intestinal con anastomosis primaria o derivación, si bien esto no suele ser preciso.

Entre las técnicas para prevenir la recurrencia de la herniación encontramos el cierre del hiato de Winslow (método controvertido por el riesgo de desarrollar trombosis de la vena porta)^14^, la fijación del ciego y colon ascendente o incluso algunos grupos plantean la hemicolectomía derecha^15^. Ninguna de estas técnicas está estandarizada en la práctica clínica, no existiendo consenso acerca de su utilidad.

El pronóstico a largo plazo de estos pacientes es bueno. No obstante, la presentación inespecífica del cuadro, junto con la baja prevalencia de este grupo de patologías, pueden retrasar el diagnóstico de hernias internas abdominales a través del hiato de Winslow, aumentando su comorbilidad. Por ello, es importante tener en cuenta esta patología en el diagnóstico diferencial de pacientes con un cuadro de obstrucción intestinal sin antecedentes quirúrgicos previos.
